# CAR‐T cells in the treatment of multiple myeloma: an encouraging cell therapy

**DOI:** 10.3389/fimmu.2025.1499590

**Published:** 2025-02-26

**Authors:** Tong Yu, Jian-Hang Jiao, Min-Fei Wu

**Affiliations:** Department of Orthopaedic Medical Center, The Second Norman Bethune Hospital of Jilin University, Changchun, Jilin, China

**Keywords:** multiple myeloma, CAR, T cell, immunotherapy, CAR-T

## Abstract

Multiple myeloma (MM) is a malignant disease of plasma cells that accounts for approximately 10% of all hematological malignancies and is characterized by a clonal proliferation of malignant plasma cells in the bone marrow. Numerous therapeutic strategies, including proteasome inhibitors, immunomodulators, monoclonal antibodies against CD38 and autologous stem cell transplantation, have prolonged the median survival of MM patients. Nevertheless, almost all MM patients suffer disease relapses due to drug resistance and eventually die from MM or MM-related complications. Chimeric antigen receptor (CAR) T-cell therapy is a novel immunotherapy strategy for MM and has shown encouraging results in several clinical trials. However, the use of CAR T-cell therapy for the treatment of MM is still associated with several difficulties, including antigen escape, poor persistence, an immunosuppressive microenvironment, cytokine release syndrome, immune effector cell-associated neurotoxicity syndrome, CAR T-cell-associated encephalopathy syndrome, cytopenia, and infections. In this review, we describe in detail the target antigens of CAR T cells in MM. We also comprehensively discuss recent innovations in the development of CAR T cells to improve clinical efficacy and strategies to overcome the limitations of CAR T-cell therapy in MM.

## 1 Introduction

Multiple myeloma (MM) is a malignant disease of plasma cells that accounts for approximately 10% of all hematological malignancies (1). It is characterized by clonal proliferation of malignant plasma cells in the bone marrow associated with the overproduction of monoclonal immunoglobulin protein (known as M protein) and subsequent damage to internal organs (2). The main clinical manifestations of MM are osteoporosis, hypercalcemia, bone pain and pathological fractures. In the past, many therapeutics, including proteasome inhibitors, immunomodulators and monoclonal antibodies to CD38, have extended the median survival of MM patients from 3 to 6 years after initial diagnosis (3). Unfortunately, almost all MM patients eventually relapse (4). Moreover, with each new MM relapse, malignant plasma cells undergo clonal evolution and acquire new mutations that lead to disease progression and resistance to conventional treatments (5). In 2020, cancer statistics in the United States showed an MM related mortality rate of 39.76% (6). Therefore, new therapeutic approaches for the treatment of MM are urgently needed.

Chimeric antigen receptor (CAR) T cell therapy mediate tumor killing in several ways, including secretion of cytotoxic granules containing perforin and granzymes, production of pro-inflammatory cytokines such as IFN-g and TNF-a and activation of Fas/Fas ligand (Fas/FasL), elevation of circulating levels of serum cytokines (such as IL-15) and depletion of endogenous regulatory T cells (7). In recent years, CAR T cell therapy has been widely used to treat a number of malignancies, including MM, leukemia, ovarian cancer, breast cancer and osteosarcomas (8, 9). However, the clinical application of CAR T cell therapy for MM still faces several disadvantages, including antigen escape, poor persistence and an immunosuppressive microenvironment, cytokine release syndrome (CRS), immune effector cell-associated neurotoxicity syndrome (ICANS) and CAR T cell-related encephalopathy syndrome (10). In this review, we therefore describe in detail the structure of CAR T cells, the target antigens of CAR T cells in MM, the dilemmas of CAR T cell therapy for MM and the ways to solve these dilemmas.

## 2 CAR T cells

CAR is a genetically engineered chimeric receptor consisting of an extracellular single-chain variable fragment (scFv), a hinge region, a transmembrane structural domain, an intracellular costimulatory structural domain and a CD3ζ activating structural domain, which enables T cells to reorient towards target antigens, thereby producing a cytotoxic effect that is completely independent of the expression of the major histocompatibility complex of the target cell (11). In particular, scFv is a fusion protein consisting of variable regions, such as the heavy and light chains in monoclonal antibodies, which are responsible for recognizing specific antigens on the surface of tumor cells. The hinge region serves as a bridge connecting scFv to the transmembrane structural domain. The transmembrane structural domains have the ability to anchor CAR to the T cell membrane and facilitate mechanical signal transduction into the cell (12).Costimulatory structural domains include CD28, 4-1BB, ICOS and OX40; of these, CD28 and 4-1BB are the most commonly used costimulatory structural domains. CD3ζ is the most commonly used activation structural domain. It is located in the innermost part of the CAR and is able to trigger moderate secretion of IL-2, which plays a role in promoting T cell activation and tumor cell cleavage (13).

**Figure 1:**
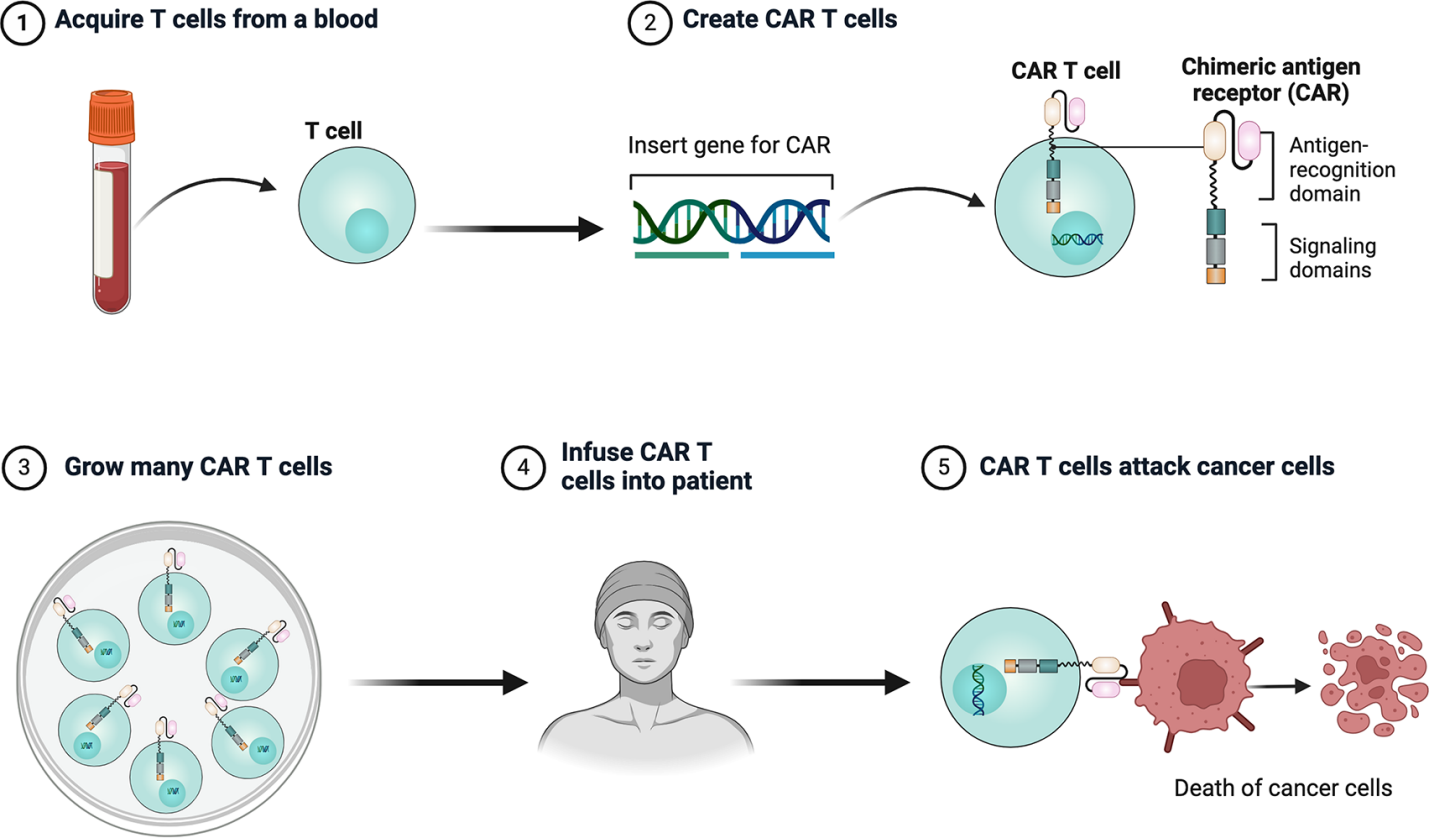
Workflow of CAR T-cell lysis of tumor cells.

**Figure 2:**
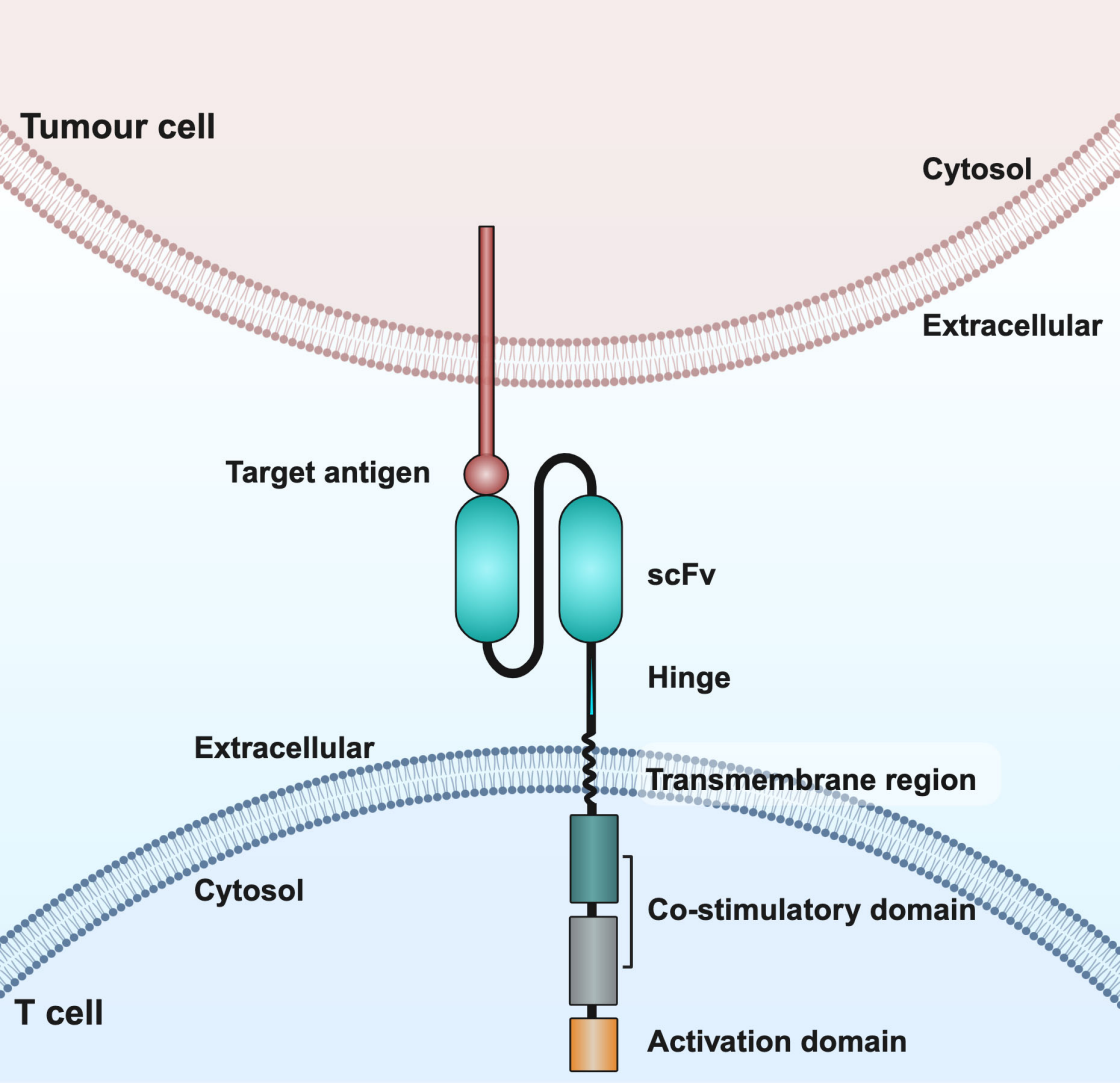
Schematic structure of CAR T cells. CAR consists of an extracellular structural domain, a transmembrane region, and an intracellular structural domain. scFv, single-chain variable fragment.

CAR T cell therapy involves modifying a patient’s own T cells to better recognize and attack cancer cells. First, T cells are collected from the patient, then genetically engineered in the lab to express a CAR that targets cancer cell markers. These modified T cells are then multiplied and infused back into the patient, where they target cancer cells by recognizing the target antigen (**Figures 1**, **2**).

## 3 Target antigens

**Figure 3:**
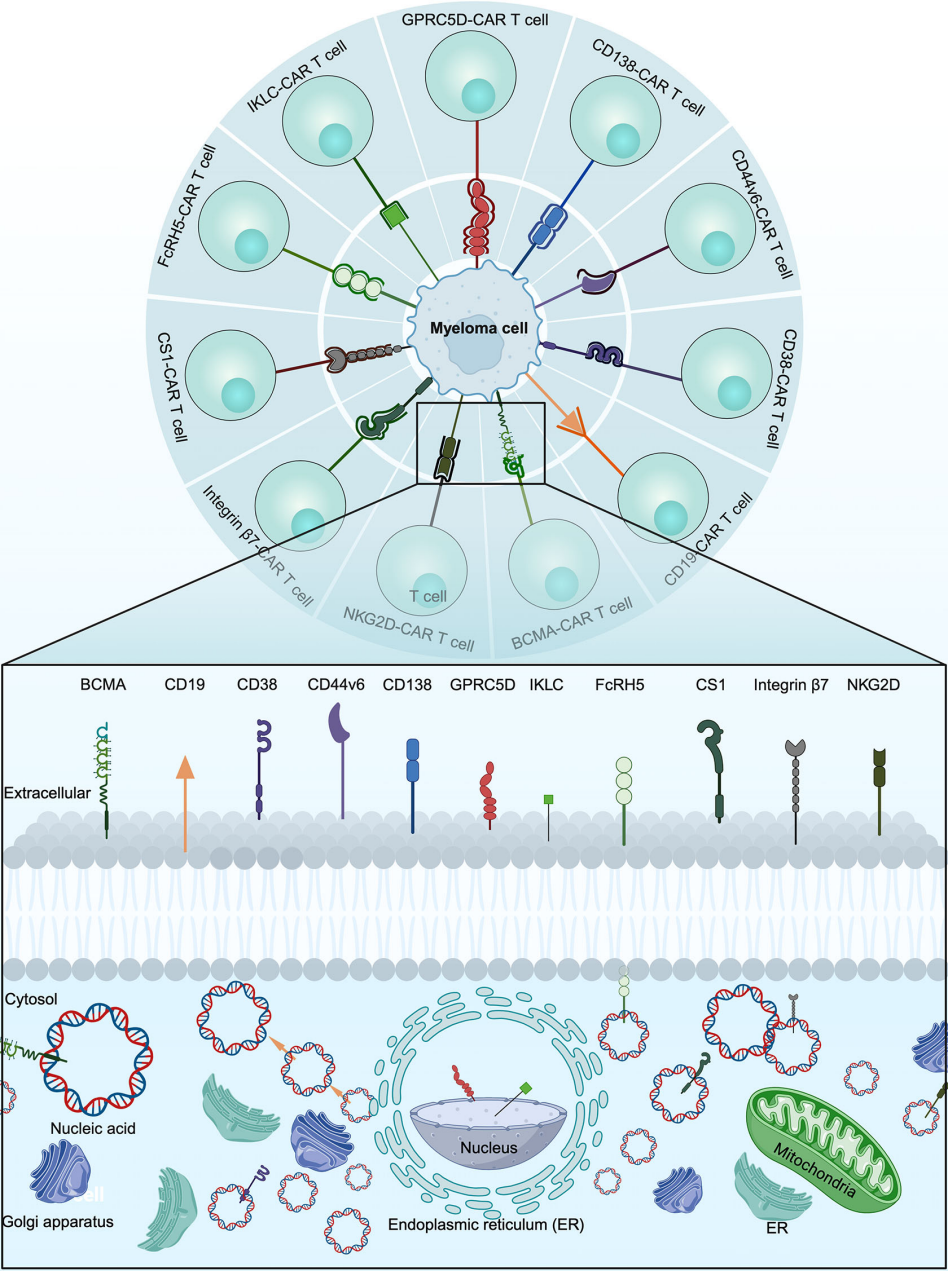
The main targets of CAR T cells for the treatment of MM.

The most important determinant for the success of CAR T cell therapy is the choice of target antigen (14). The optimal CAR T cell target antigen is one that is consistently expressed on the surface of malignant cells but not on the surface of non-malignant cells (15). A variety of target antigens, including B-cell maturation antigen (BCMA), CD19, CD38, CD44v6, CD138, G protein‐coupled receptor, class C, group 5, member D (GPRC5D), immunoglobulin kappa light chain (IKLC), Fc receptor-homologue 5 (FcRH5), signaling lymphocyte activation molecule member 7 (SLAMF7), integrin β7 and natural killer group 2 member D (NKG2D), were investigated for the treatment of MM (**Figure 3**).

### 3.1 BCMA

BCMA is a cell surface protein expressed mainly on plasma cells, mature B lymphocytes and most MM cells (16). BCMA is the most attractive target antigen for CAR T cell therapy for MM, and BCMA-CAR T cell therapy has achieved unprecedented responses in recurrent or refractory MM patients, bringing new hope for these patients (17). In untreated and relapsed MM patients, the expression rate of BCMA on the surface of tumor cells was 100% (18). Currently, two BCMA-CAR T cell products, idecabtagene vicleucel (ide-cel) (19) and ciltacabtagene autoleucel (cilta-cel) (9), have been approved by the US Food and Drug Administration (FDA) for the treatment of recurrent or refractory MM.

Cilta-cel is a CAR T cell that expresses two antigen recognition regions that target BCMA. A phase II clinical trial showed that 72% of patients showed a durable antitumor response after infusion of cilta-cel (9). A phase III, randomized, open-label trial found that a single cilta-cel infusion resulted in a lower risk of disease progression or death than standard care in lenalidomide-refractory patients with MM who had received one to three previous therapies (NCT04181827) (20).

A phase II clinical trial showed that 73% of patients with relapsed and refractory myeloma were effectively treated with ide-cel, and 33% achieved complete remission (19). Recently, an international, open-label, phase 3 trial reported that ide-cel therapy significantly prolonged progression-free survival and improved response as compared with standard regimens in patients with triple-class-exposed relapsed or refractory MM who had received two to four regimens (including immunomodulatory agents, proteasome inhibitors, and daratumumab) previously. The toxicity of ide-cel was consistent with previous reports (NCT03651128) (21).

BCMA-CAR T cell therapy has demonstrated robust therapeutic activity in clinical trials, but toxicities remain a significant concern for a subset of patients. Thus, fully humanized scFv, which reduces immunogenicity, was developed. Researchers developed a fully human BCMA-specific CAR, CT103A, to improve clinical efficacy (22). In a phase 1 trial, CT103A was found to be safe and highly active in patients with RRMM, even at lower doses (1 and 3 × 10^6^ CAR+ T cells/kg) with minimal side effects, including CRS and neurotoxicity (22). Patients who relapsed after prior murine BCMA CAR T cell therapy also benefited from CT103A (22). In a phase 1 clinical trials, Yang et al. generated CT053 with an optimized, fully-human scFv, and demonstrated that CT053 had strong efficacy and a good safety profile when administered to relapsed or refractory MM patients (23). Moreover, to enhance CAR T cell therapy and overcome PD-L1-mediated suppression, Pornpimon et al. developed anti-BCMA-CAR5-T cells, which incorporate three costimulatory domains and the ability to secrete anti-PD-L1 scFv. These cells were generated by fusing anti-BCMA-CAR4-T cells, which contain a human anti-BCMA scFv and costimulatory domains (CD28, 4-1BB, CD27) linked to CD3ζ, with anti-PD-L1 scFv. Anti-BCMA-CAR5-T cells demonstrated specific cytotoxicity against BCMA-expressing targets, enhanced CAR-T cell proliferation, and sustained CAR expression after antigen re-stimulation. Additionally, they lowered PD-1 levels and reduced PD-L1 expression on target cells, resulting in improved antitumor activity, better proliferation, and reduced T-cell exhaustion in MM models (24). Taken together, BCMA is a potential and promising target antigen for CAR-T cell therapy for MM.

### 3.2 CD19

CD19 is a B lymphocyte-specific surface antigen that belongs to the immunoglobulin superfamily and is predominantly expressed in mature cells of the B-cell lineage (25). Although only a small proportion of plasma cells express CD19, importantly, studies have found that CD19+ multiple myeloma patients have a poor prognosis, characterized by a tendency to relapse and short survival (26). An early phase study by Garfall et al. found that ten patients received CD19-CAR T cells after high‐dose melphalan and autologous stem cell transplantation, and two patients had significantly longer PFS with CD19-CAR T cells plus autologous stem cell transplantation compared to their previous autologous stem cell transplantation (27). Therefore, they concluded that CD19-CAR T cells may improve the duration of response to standard therapies for MM by targeting and eliciting secondary immune responses against myeloma-spreading cells (27). In addition, Shi et al. reported the results of a phase 1/2 study of bispecific CAR T cells targeting CD19 and BCMA in relapsed or refractory MM. The study demonstrated that bispecific CD19-BCMA CAR T cells were feasible, safe, and effective in patients with relapsed or refractory MM. Future prospective and multicenter clinical trials with larger sample sizes and longer follow-up are needed to evaluate the long-term impact of CD19-BCMA CAR T cell therapy on patient outcomes (28). Thus, CD19 is a potential, promising, valuable and important target antigen for CAR T cell therapy for MM.

### 3.3 CD38

CD38 is a transmembrane glycoprotein associated with calcium regulation, signal transduction and cell adhesion (29). More than 90% of MM patients express CD38 on malignant plasma cells (30). Daratumumab and isatuximab are two CD 38 antibodies which FDA approved by FDA (31). Preclinical evidence that CD38-CAR T cells are effective in the treatment of myeloma (32). Besides, CD38-CAR T cells are being investigated in an ongoing clinical trial (NCT03464916). A phase 1/2 trial is investigating dual-targeted tandem CD38‐BCMA-CAR T cells in recurrent or refractory MM (NCT03767751) (33). In addition, studies have also shown the downregulation of CD38 expression in recurrent or refractory MM, raising concerns about adverse resistance (34). However, it is undeniable that CD38 is a potential and promising target antigen for CAR T cell therapy for MM.

### 3.4 CD44v6

CD44, the hyaluronan receptor, is a class I membrane glycoprotein that is overexpressed in hematological and epithelial tumors and can be expressed in both the canonical form (CD44s) and a variety of isoforms (CD44v), which include CD44v6 (35). CD44v6 expression correlates with chromosomal band 13q14 deletions, a well-known risk factor in MM. CD44v6 is frequently expressed in advanced, high-risk MM, and CD44v6 positivity is seen in 17% of stage I leukemia MM and 43% of stage II/III leukemia MM or plasma cell leukemia (36). Moreover, Monica et al. found that CD44v6-CAR T cells have an inhibitory effect on myeloma cell proliferation without damage to hematopoietic stem cells, because CD44v6 is essential for myeloma cell growth (37). Thus, CD44v6 is a potential and promising target antigen for CAR T cell therapy for MM.

### 3.5 CD138

CD138 is a known cell surface antigen on myeloma cells, and its expression has been positively associated with plasma cell proliferation and survival in MM (38). CD138 is also highly expressed in normal tissues, raising concerns that anti‐CD138 therapies may be toxic outside the tumor. In the phase 1 dose‐escalation study (NCT01886976), autologous CD138-CAR T cell monotherapy was used to treat five patients with recurrent or refractory MM. In four of them, the disease remained stable for more than three months, and in one advanced-stage patient, the number of myeloma cells in the peripheral blood decreased from 10.5% to < 3%. No patient showed a complete response, and no intolerable toxic effects were observed during treatment (39). Thus, CAR T cell therapy targeting CD138 is safe and feasible. It has potential antitumor activity *in vivo*, but the sample size of this clinical trial was too small. Thus, further studies with CD138-CAR T cell therapy for MM are needed to fully evaluate its effects.

### 3.6 GPRC5D

GPRC5D has emerged as a novel target for CAR T‐cell therapy in recurrent or refractory MM. GPRC5D expression is restricted to malignant plasma cells and hair follicles, making it an attractive therapeutic target (40). MCARH109, a novel CAR T cell construct targeting GPRC5D, showed promising efficacy in a phase 1 trial in patients with heavily treated recurrent or refractory MM, even in those who had previously progressed on BCMA‐targeted therapy. At the time of the last update in September 2022, a total of 17 patients had received MCARH109, 12 of whom received the maximum tolerated dose. Of these, seven patients (58%) had a partial response or better, with three patients (25%) achieving complete remission. Of note, of the six patients who had previously received BCMA therapy, three patients (50%) achieved a partial response or better. With a median follow‐up of 10.1 months, six of the 12 patients (50%) with a partial response or better remained progression-free (40). An updated analysis of the phase 1 trial of MCARH109 (GPRC5D-CAR T cell therapy for MM, NCT04555551) showed no new serious adverse events at a median follow-up of 37 months, with 71% of patients responding, a median response duration of 8.6 months, a 3-year overall survival estimate of 59%, and an association between an activated T-cell phenotype at apheresis and response, while possible GPRC5D loss was observed in 60% of patients at relapse (41). A phase 2, open label, multicenter study of BMS-986393, a GPRC5D-CAR T Cell therapy in adult participants with relapsed or refractory MM (QUINTESSENTIAL) (NCT06297226) is ongoing. Although existing clinical trial studies targeting the GPRC5D antigen have small sample sizes, they have also demonstrated its feasibility as an MM antigen target, making GPRC5D-CAR T cells a promising treatment for MM patients.

### 3.7 κ-light chain

Only mature B cells and malignant cells derived from mature B cells express surface immunoglobulins with a κ-light chain. Therefore, CAR T cells targeting immunoglobulin light chains (κ or λ) have been used to lyse MM cells expressing a specific light chain while avoiding cytotoxicity to healthy mature B cells expressing other light chains (42). Ramos et al. developed a second-generation CAR T cell (κ-CAR T) that targets the κ-light chain, and a phase 1 clinical trial showed that four of seven MM patients responded to infusion of κ-CAR T cells and achieved disease stabilization for more than 24 months or overall improvement in disease symptoms. Moreover, the CAR T cell product was well tolerated, with no patients experiencing severe CRS (43). Thus, CAR T cells targeting the immunoglobulin κ light chain are a safe and effective treatment for patients with relapsed or refractory MM.

### 3.8 FcRH5

FcRH5 can first be detected in progenitor B cells and is maintained on normal plasma cells (44). However, FcRH5 expression is upregulated on malignant plasma cells in MM compared to normal plasma cells. Jiang et al. found in a preclinical study that FcRH5 CAR T cells triggered antigen-specific activation, cytokine secretion and cytotoxicity against MM cells. Bispecific FcRH5-BCMA-CAR T cells efficiently recognized MM cells expressing FcRH5 and/or BCMA and showed enhanced efficacy compared to monospecific CAR T cells (45). In addition, cevostamab is a bispecific FcRH5-CD3 antibody that facilitates the T cell-directed killing of myeloma cells. The phase 1 clinical trial (NCT03275103) showed that cevostamab monotherapy in patients with heavily pretreated relapsed or refractory MM continued to have clinically meaningful activity and manageable safety without seriously damaging vital organs or tissues (46). Thus, FcRH5-CAR T cells may represent a promising therapeutic avenue for MM.

### 3.9 CS1/SLAM7

CS1 is a cell surface glycoprotein of the lymphocyte signaling activation molecule F7 (SLAM7) family that is strongly expressed on myeloma cells and weakly expressed in normal tissue cells (47). To confirm the feasibility of CS1-CAR T cells for the treatment of MM, researchers examined the effect of CS1-CAR T cells on various MM cells, and the data showed that CS1-CAR T cells had antigen-dependent killing effects. Interestingly, CS1-CAR T cells had a beneficial therapeutic effect in dexamethasone-resistant MM patients (48). It is also the target of the fully humanized elotuzumab, which is currently FDA-approved for the treatment of recurrent or refractory MM. Preclinical data on CS1-CAR T cell therapy have shown superior efficacy compared to elotuzumab (49), and it is currently being investigated in clinical trials (NCT03958656 and NCT03710421). In Europe, the phase 1/2 trial CARAMBA (EudraCT identifier 2019‐001264‐30) is investigating a novel CS1-CAR made with virus‐free, advanced Sleeping Beauty Transposon technology, and the results are eagerly awaited (50).

### 3.10 Integrin β_7_

Integrins play an important role in the establishment, survival, proliferation and drug resistance of MM cells in the bone marrow (51). Hosen et al. found that activated integrin β_7_ was highly expressed in the cells of patients with MM, even when they had received treatment. These results suggest that CAR T cell therapies targeting activated integrin β_7_ have the potential to benefit patients with relapsed or refractory MM (52). Hosen et al. screened more than 10,000 monoclonal antibody clones against MM and identified MMG49 as an MM-specific monoclonal antibody that specifically recognizes a subset of integrin β_7_ molecules. The MMG49 epitope in the N-terminal region of the β_7_-chain is presumably inaccessible in the resting integrin conformation but exposed in the active conformation. Increased expression and constitutive activation of integrin β_7_ resulted in high MMG49 reactivity on MM cells, whereas MMG49 binding was barely detectable in other cell types, including normal integrin β_7_^+^ lymphocytes. T cells transduced with MMG49-derived CAR exerted anti-MM activity without damaging normal hematopoietic cells (53). Thus, MMG49-CAR T cell therapy is promising for MM, and a receptor protein with a rare but physiologically relevant conformation may serve as a target for cancer immunotherapy.

### 3.11 NKG2D

NKG2D is an activating receptor expressed on NK cells, invariant NKT cells, γδ T cells, CD8^+^ T cells and a small fraction of CD4^+^ T cells. NKG2D ligands are expressed on MM but not on healthy tissue (54). In addition, NKG2D ligands are also expressed on immunosuppressive cells, such as regulatory T cells and myeloid-derived suppressor cells, making NKG2D-CAR T cell therapy an attractive option in hematological malignancies and solid tumors (55). Importantly, human NKG2D-CAR T cells do not respond *in vitro* to autologous peripheral blood mononuclear cells or bone marrow from healthy donors (56). Studies by Sentman and colleagues in mice demonstrated the efficacy of NKG2D-CAR T cells in eradicating established MM, lymphomas and ovarian cancers and inducing autologous immunity that protects against tumor rechallenge even after NKG2D-CAR T cells were no longer detectable (57). The phase 1 study by Baumeister et al. showed that the produced NKG2D-CAR T cells have functional activity against autologous tumor cells *in vitro*. However, to increase clinical activity, modifications may be required to increase CAR T cell expansion and target density (57). In addition, CYAD-01 is an autologous CAR T cell product based on the NKG2D receptor, which binds eight ligands overexpressed in a variety of hematological malignancies but is largely absent on nonneoplastic cells. A phase 1 clinical trial (NCT03018405) showed that treatment with a CYAD-01 multiple infusion regimen is well tolerated without preconditioning, albeit without durability outside of patients being converted to allogeneic hematopoietic stem cell transplantation (56). These phases 1 data support the proof of concept of targeting NKG2D ligands with CAR T cell therapy. Further clinical trials with NKG2D-CAR T cells are warranted, possibly via combinatorial antigen-targeted approaches to enhance antitumor activity.

### 3.12 Others

Many clinical trials exploring the safety and efficacy of CAR T cell therapy for MM are currently either underway or completed (**Table 1**) (**Supplementary File 1**).

**Table 1:** Selected landmark clinical trials of CAR T‐cell therapy in MM.

References	Identifier	Name	Phase	Study details (at the time of reporting)	Antigen	Co-signaling domain	Dosage	Efficacy	Toxicity	Survival outcomes
Munshi et al. (19)	NCT03361748	Ide-cel KARMMA	2	N=128	BCMA	CD137	150-450×10^6^ (CAR+) T cells/kg	ORR (73%) CR (33%)	CRS (84%) Neurotoxic effects (18%)	Median PFS was 8.8 months
Jesus et al. (9)	NCT03548207	Cilta-cel CARTITUDE- 1	1b/2	N=97	BCMA	CD3ζ and 4-1BB	0.75×10^6^ (CAR+) T cells/kg	ORR (97%) sCR (67%)	CRS (95%) Neutropenia (95%) Anaemia (68%) Leukopenia (61%) Thrombocytopenia (60%) Lymphopenia (50%) Neurotoxicity (21%) Deaths (14%)	12-month PFS rate was 77%; OS rate was 89%
Costello et al. (58)	NCT03288493	bb21217 CRB- 402	1/2	N=69	BCMA	4-1BB	150-450×10^6^ (CAR+) T cells/kg	ORR (60%) CR (28%)	CRS (70%) ICANS (16%)	–
Van et al. (59)	NCT03548207	Orva-cel EVOLVE	1/2	N=62	BCMA	4-1BB	150-600×10^6^ (CAR+) T cells/kg	ORR (92%) CR (36%)	CRS (89%) ICANS (13%)	–
Syed et al. (60)	NCT02215967	CAR-BCMA	1	N=12	BCMA	CD28 and CD3ζ	0.3–9×10^6^ (CAR+) T cells/kg	sCR (8%); VGPR (17%); PR (8%); SD (67%)	CRS (17%) Other toxicities (83%)	–
Mailankody et al. (61)	NCT03070327	MCARH171	1	N=11	BCMA	–	72–818×10^6^ (CAR+) T cells/kg	VGPR: 2; ORR: 64%	CRS (60%)	–
Raje et al. (62)	NCT02658929	CRB401	1	N=33	BCMA	4-1BB	50-450×10^6^ (CAR+) T cells/kg	ORR 85%	CRS (76%)	Median PFS was 11.8 months
Popat et al. (63)	NCT03287804	AUTO2	1/2	N=11	BCMA+TACI	CD28-OX40	15-900×10^6^ (CAR+) T cells/kg	ORR (43%)	CRS (45%)	–
Garfall et al. (27)	NCT02135406	CTL019	1	N=10	CD19	CD3ζ and 4-1BB	140-200 (mg/m^2^) ASCT+CTL019	VGPR (60%) PD (20%) PR (20%)	Autologous graft-versus-host disease (gastrointestinal) (10%) Mucositis (10%)	Median PFS was 6.6 months
Mailankody et al. (40)	NCT04555551	MCARH109	1	N=17	GPRC5D	CD3ζ and 4-1BB	25-450×10^6^(CAR+) T cells/kg	ORR (71%) CR (35%) PR (71%)	CRS (6%) Cerebellar disorder (12%)	Median PFS was 7.8 months
Ramos (43)	NCT00881920	κ-CAR T	1	N=7	κ light chain	CD28 and ζ	0.092-1.8×10^8^ cells/m^2^	ORR (57%)	No	–
Trudel et al. (46)	NCT03275103	Cevostamab	1	N=160	FcRH5xCD3 bispecific antibody	–	single step-up D1:0.05-3.6mg, D8: 0.15-198mg, double step-up D1: 0.3-1.2mg, D8: 3.6mg, D15: 60-160mg	ORR (54.5%)	CRS (80%) Infections (42.5%) Neurological/psychiatric (40.6%) Anemia (31.9%) Diarrhea (26.3%)	Median duration of response was 15.6 months
Prommersberger et al. (50)	EudraCT: 2019-001264-30	CARAMBA	I/IIA	N=38	SLAMF7/CS1	CD28	–	–	–	–
Baumeister et al. (57)	NCT02203825	NKG2D-CAR T cells	1	N=5	NKG2D	CD3ζ	1-30×10^6^ cells/kg	–	CRS (0%) ICANS (0%)	Median OS was 4.7 months
Guo et al. (39)	NCT01886976	CART-138	1	N=5	CD138	4-1BB	Mean 7.6×10^8^ (CAR+) T cells/kg	–	CRS (80%)	–

CRS, cytokine release syndrome; ORR, overall response rate; sCR, stringent complete response; CR, complete response; VGPR, very good partial response; PR, partial response; PD, progression of disease; PFS, progression-free survival; OS, overall survival; transmembrane activator and calcium-modulator and cyclophilin ligand interactor (TACI); ASCT, autologous stem cell transplantation.

Pediatric MM is rare with only approximately 0.3% of cases diagnosed before the age of 30 (64). CAR T cell therapy for pediatric MM is an emerging treatment with ongoing research focused on addressing the unique challenges in children. Key targets for CAR T cells include BCMA, GPRC5D, and CD38, with the aim of improving the targeting and killing of malignant plasma cells. However, pediatric MM presents with more aggressive disease characteristics, and the immune system in children may respond differently to treatment, requiring tailored protocols for safety. Further research is needed to refine these therapies for pediatric patients and enhance long-term outcomes.

The success of CAR T-cell therapy has led researchers to explore engineering other immune cells, such as natural killer (NK) cells, NKT cells (65), macrophages (66), and neutrophils (67), for therapeutic use. CAR-NK cell therapy, in particular, has shown promising results in clinical trials. While these immune cells may have fewer concerns regarding graft-versus-host disease, making them potential off-the-shelf products, they face limitations like short lifespans, limited proliferation, and an inability to form memory cells. Additionally, T cells can be engineered to target tumors using tumor-neoantigen-specific TCRs (68), offering an advantage over CARs by targeting not only membrane antigens but also intracellular neoantigens in the context of MHC complexes (69).

## 4 Challenges

### 4.1 CAR preparation failure

**Figure 4:**
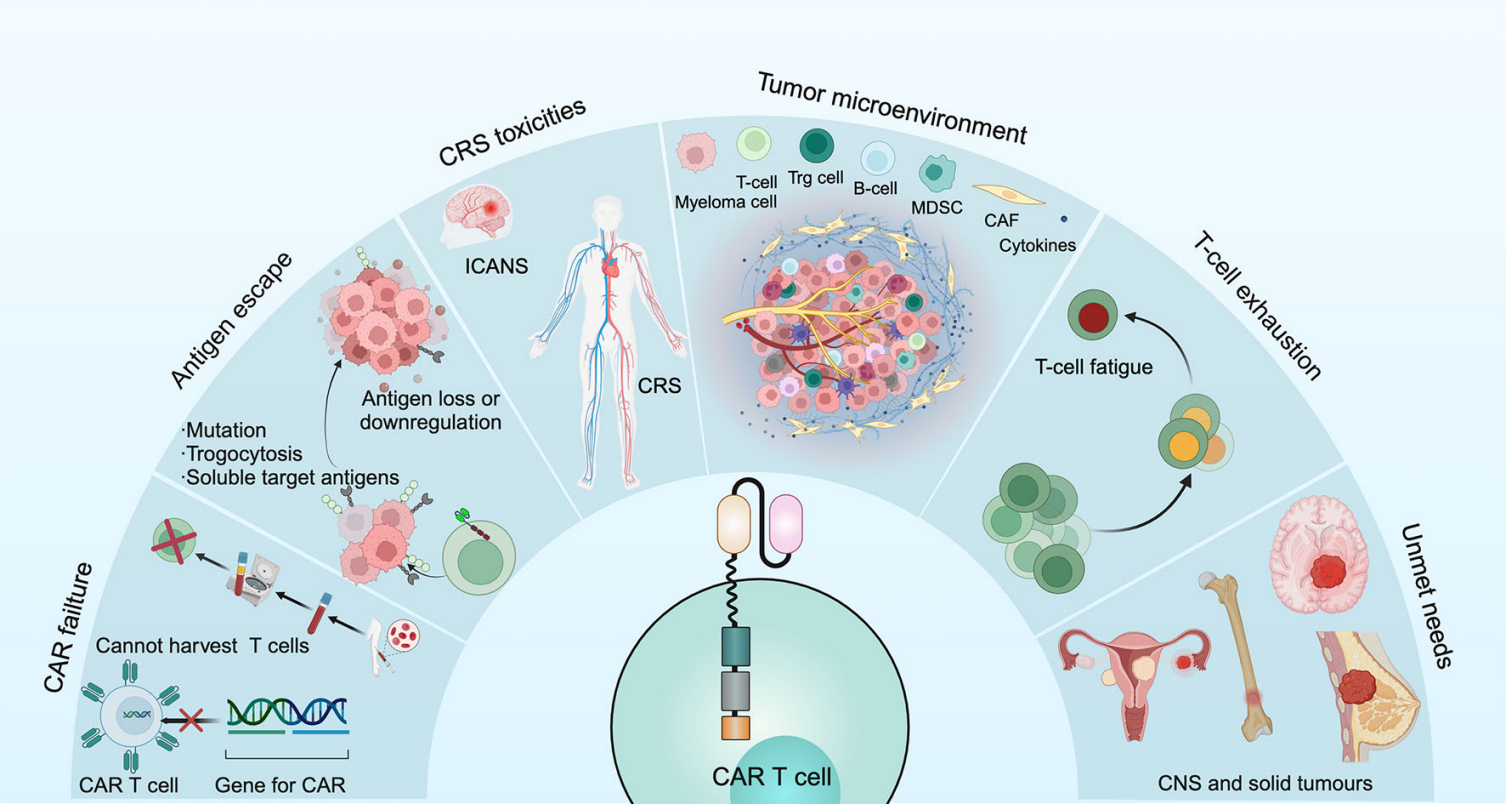
Current dilemmas of CAR T cell therapy for the treatment of MM.

CAR T cell failures have several causes: for some patients, the CAR T cell product cannot be successfully manufactured or the generated CAR T cells do not expand sufficiently (either during manufacturing *in vitro* or after administration *in vivo*); in other patients, the problem of limited persistence *in vivo* is a potential mechanism underlying disease relapse (70) (**Figure 4**).

### 4.2 Tumor antigen escape

Myeloma cells can undergo loss or downregulation of target antigens, which occurs through 2 main mechanisms. One mechanism is that T cells can actively transfer antigens from MM cells to T cells through trogocytosis, which not only downregulates the number of target antigens on MM cells but also promotes T cell cytotoxicity (71). Another mechanism is the cleavage of BCMA by γ-secretase in myeloma cells, leading to a short extracellular structural domain of BCMA allowing direct shedding and an increase in soluble BCMA, thus binding these antigens to CAR T cells and limiting their effectiveness (72) (**Figure 4**).

### 4.3 CRS and ICANS

Roex et al. investigated the safety of CAR T cell therapy in 639 patients with MM and found that the incidences of CRS and ICANS were 80.3% and 10.5%, respectively (73).

CRS is a systemic inflammatory response syndrome triggered by T cell activation and mediated by a variety of cytokines, such as IFN-γ, TNF-α, IL-8, IL-10 and macrophage colony-stimulating factor. Common symptoms include fever, chills, tachycardia, tachypnoea, myalgia, and it can lead to hypoxia, cardiac arrhythmias, renal failure, liver damage, coagulopathy and multiorgan failure (74). Morgan et al. reported serious adverse events, even fatal, caused by CRS (75). According to the CRS grading system of Lee et al, the toxicity of CRS is classified as 1-5 from mild to severe (74). The treatment of CRS varies significantly based on severity. Mild CRS (Grade 1 or 2) is managed with supportive care, including hydration, antipyretics, and monitoring. Moderate CRS (Grade 2) is treated with tocilizumab, which blocks IL-6, along with supportive care as needed. Severe CRS (Grade 3 or 4) requires higher doses or repeated tocilizumab, corticosteroids like dexamethasone, and intensive care, including mechanical ventilation and organ support (22, 63, 76). Pre-emptive tocilizumab may be used for high-risk patients, and early detection and intervention are crucial for improving outcomes.

ICANS is a neurological complication that can occur as a result of certain immunotherapies, particularly CAR T-cell therapy and other adoptive T-cell therapies. It is considered one of the key complications associated with these treatments, and its severity can range from mild to life-threatening. The severity of ICANS was dose- and tumor burden-dependent and correlated positively with the severity of CRS (33) (**Figure 4**). ICANS occurs when CAR T-cells, while targeting and destroying cancer cells, release inflammatory cytokines such as IL-6 and other immune mediators. These substances not only affect the tumor but also impact the brain and nervous system, leading to neurotoxicity (77). The presentation of ICANS can vary widely but commonly includes cognitive impairment, weakness or paralysis, seizures, speech difficulties, headache, and changes in behavior (78). ICANS is classified by severity from grade 1 to grade 4. Grade 1 involves mild confusion and transient neurological symptoms; grade 2 includes noticeable confusion or agitation without major functional impairment; grade 3 consists of severe symptoms like confusion, disorientation, or seizures that affect daily activities; grade 4 represents life-threatening conditions, such as coma or continuous seizures, requiring intensive care (79). Risk factors for ICANS include severe CRS, older age, pre-existing neurological conditions, and certain CAR T-cell characteristics (2, 10, 80). Treatment focuses on controlling inflammation and managing symptoms with corticosteroids, tocilizumab, supportive care, and symptomatic treatment (71). The prognosis varies; mild to moderate cases often resolve with intervention, but severe cases may result in long-term impairment or be life-threatening if not treated promptly.

### 4.4 Tumor microenvironment

MM has a remarkably complex bone marrow microenvironment involved in promoting tumor growth, immune escape and drug resistance (81). The bone marrow microenvironment accumulates various immunosuppressive cells, including regulatory T- and B-cells, myeloid derived suppressor cells, tumor associate macrophages, dysfunctional dendritic cells as well as mesenchymal stromal cells and osteoclasts, which can reduce the anti-MM efficacy of CAR T cells (82). Moreover, metabolism has been shown to be important for the differentiation and functional expression of T cells. In the tumor microenvironment, tumor cells compete with T cells for the use of glucose, thus suppressing T cell function (83). In addition, high concentrations of potassium ions released by dead tumor cells strongly inhibit T cell function (84). The decrease in specific nutrients and the accumulation of metabolic waste thus lead to a change in the microenvironment and adversely affect T cell function (**Figure 4**).

### 4.5 T cell exhaustion

T cell exhaustion is a critical obstacle to achieving a sustained killing effect after CAR T cell therapy. According to data from the clinical trial with ide-cel, CAR T cells were detectable up to 3 months after infusion and then began to decline. By 20 months after infusion, they were only detectable in 12% of patients (62). T cell persistence is negatively affected by a number of factors, including ongoing chemotherapy and auto-immunogenicity (85). Dancy et al. demonstrated that the composition of T cells changes over time when patients receive multiple chemotherapies. Moreover, CAR T cells can be eliminated by the patient’s own immune system due to their auto-immunogenicity (86) (**Figure 4**).

### 4.6 Unmet needs

Additionally, the outcomes of CAR T cell therapy in pediatric patients with lymphoma and in patients with central nervous system (CNS) involvement remain an area of ongoing investigation. Notably, such therapies currently have limited efficacy in patients with solid tumors, and approaches to optimize response are being explored (70).

## 5 Strategies for overcoming obstacles

### 5.1 Optimizing the CAR structure

**Figure 5:**
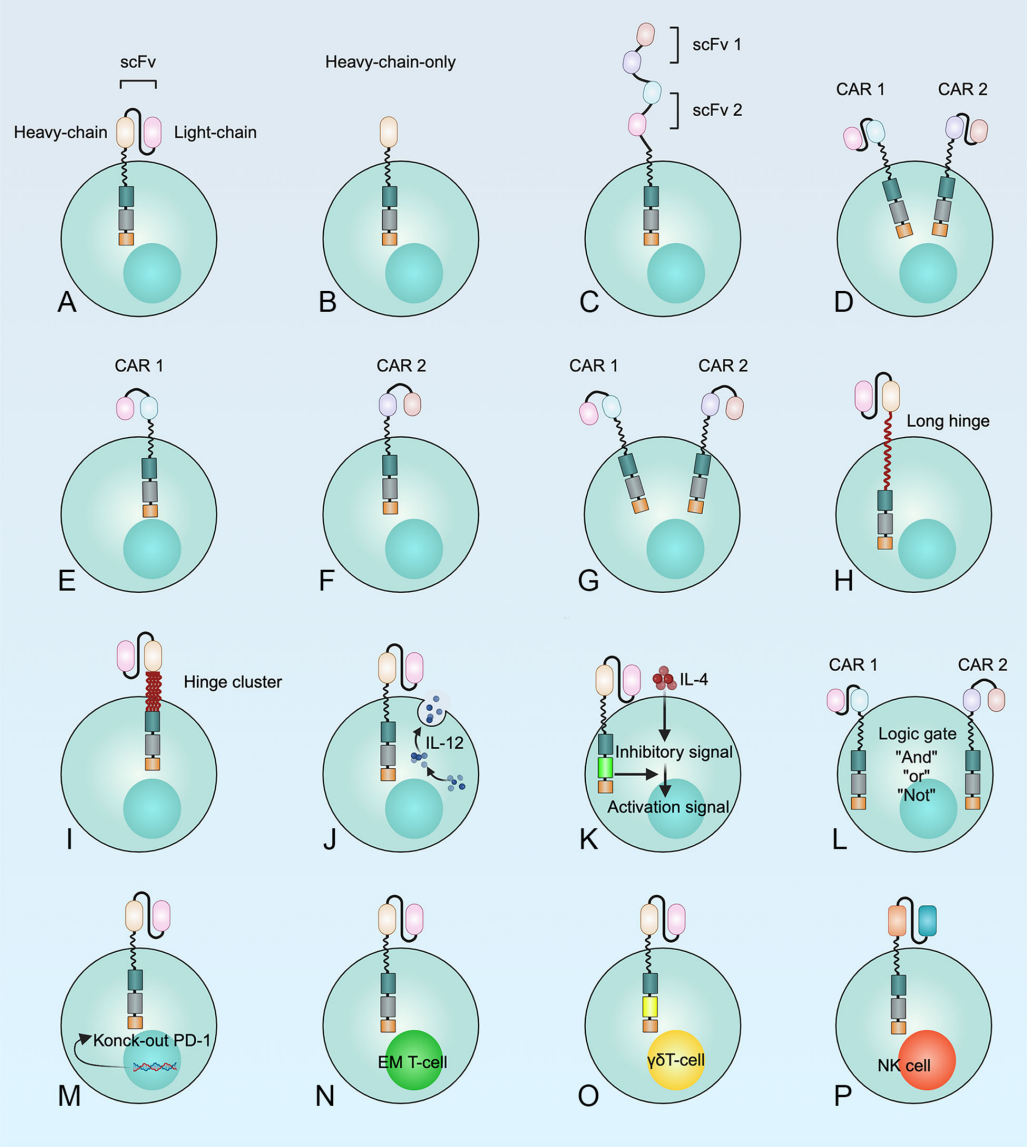
Multiple methods for the development of CAR T cell structures to optimize the therapeutic efficacy of CAR T cells in MM. **(A)** T cell expressing a monospecific CAR; **(B)** CAR T cells with only one heavy chain; **(C)** Tandem CAR T cells; **(D)** T cells transduced with a bicistronic CAR construct encoding two separate monospecific CARs, resulting in the simultaneous expression of both CARs; **(E-G)** Dual transduction describes the simultaneous transduction of an ex vivo T cell with two different CAR vectors; **(H)** Prolonged or **(I)** clustered hinge regions can improve recognition and binding of target antigens by CAR T cells; **(J)** “Armored” CAR T cells; **(K)** “Commuting” CAR T cells; **(L)** “Gated” CAR T cells. “AND-gate”, “OR-gate” and “NOT -gate” were developed to reduce the risk of “on-target, off-tumor” toxicities or "off-target" detection; **(M)** Knock-out of the immune checkpoint; **(N)** CAR early memory T cells; **(O)** CAR γδT cells; **(P)** CAR-NK cells.

Optimizing the CAR structure is an effective way to overcome antigen escape after CAR T cell therapy, which can be achieved by targeting more than one antigen on cancer cells at a time. According to the composition of CAR, scholars have developed only heavy chains CAR (87), tandem CAR (88), bicistronic CAR (89), cotransduction CAR (8), long hinges CAR (90), hinge clusters CAR (90), armored CAR (80), commutative CAR (80), knock-out CAR (80), and gated CAR (80) by engineering extracellular recognition domains, transmembrane domains, and intracellular structural domains. In addition, researchers have found that CAR T cells made from early memory T cells and γδ T cells are also an effective way to improve the function of CAR T cells (80). On the other hand, CAR-NK cells are also an effective anti-tumor immunotherapy (80) (**Figure 5**).

A phase 1 study showed that tandem therapy with BCMA-CD38-CAR T cells had an overall efficacy of 87.5% in treating patients with MM. Four patients needed tocilizumab for CRS grade 3~4, but none of them reported neurotoxicity (91). Thus, the tandem strategy with two antigens could prevent tumor recurrence. Moreover, preclinical data have shown that bispecific BCMA-CD19 CAR T cells effectively eliminate myeloma cells both *in vitro* and *in vivo*. The first human clinical trial demonstrated the superior safety and efficacy of bispecific BCMA-CD19- CAR T cells in the treatment of relapsed and refractory MM (92). Coadministration of CAR T cells is feasible in patients with relapsed or refractory MM (93). Yan et al. found that 95% of patients had remission after sequential infusion of BCMA-CAR T cells and CD19-CAR T cells in relapsed or refractory MM (93).

### 5.2 Improving safety

A perfect CAR T cell should have a strong antitumor effects but should not cause severe CRS and neurotoxic reactions. Recently, Ying et al. developed a new CD19-CAR (derived from the CD19-BBz prototype with costimulatory 4-1BB and CD3ζ structural domains) that contains longer extracellular and intracellular sequences derived from CD8α compared to conventional CD19-CAR. A clinical trial showed that CD19-BBz-CAR T cell therapy for patients with B-cell lymphoma had a complete remission rate of 54.5% (6/11) but a CRS incidence of 0% (94). These results suggest that the CAR T cell structure will continue to be optimized so that patients may not experience serious adverse effects in the future. However, further in-depth studies are needed.

### 5.3 Preventing T cell exhaustion

The long persistence of CAR T cells is an essential protection against disease relapse. CAR T cells can be administered as a series of infusions to prevent T cell exhaustion (17). Kagoya et al. placed the cytoplasmic domain of the truncated IL2 receptor-β chain and the STAT3 conjugate into a CAR construct named 28-deltaIL2RB-z (YXXQ) CAR. They found that YXXQ CAR could activate the JAK/STAT pathway when cultured with tumor antigen-positive cells and showed a longer persistence time and a more pronounced antitumor effect than conventional CAR T cells *in vivo* (95). In addition, CAR T cells produced from different subpopulations of T cells may have different proliferation abilities and persistence. In particular, CAR T cells made from early memory T cells have good proliferation ability and persistence (96). Therefore, different approaches can prolong the persistence of CAR T cells. This would improve the antitumor effect and is necessary for clinical implementation.

### 5.4 Concomitant therapies

**Figure 6:**
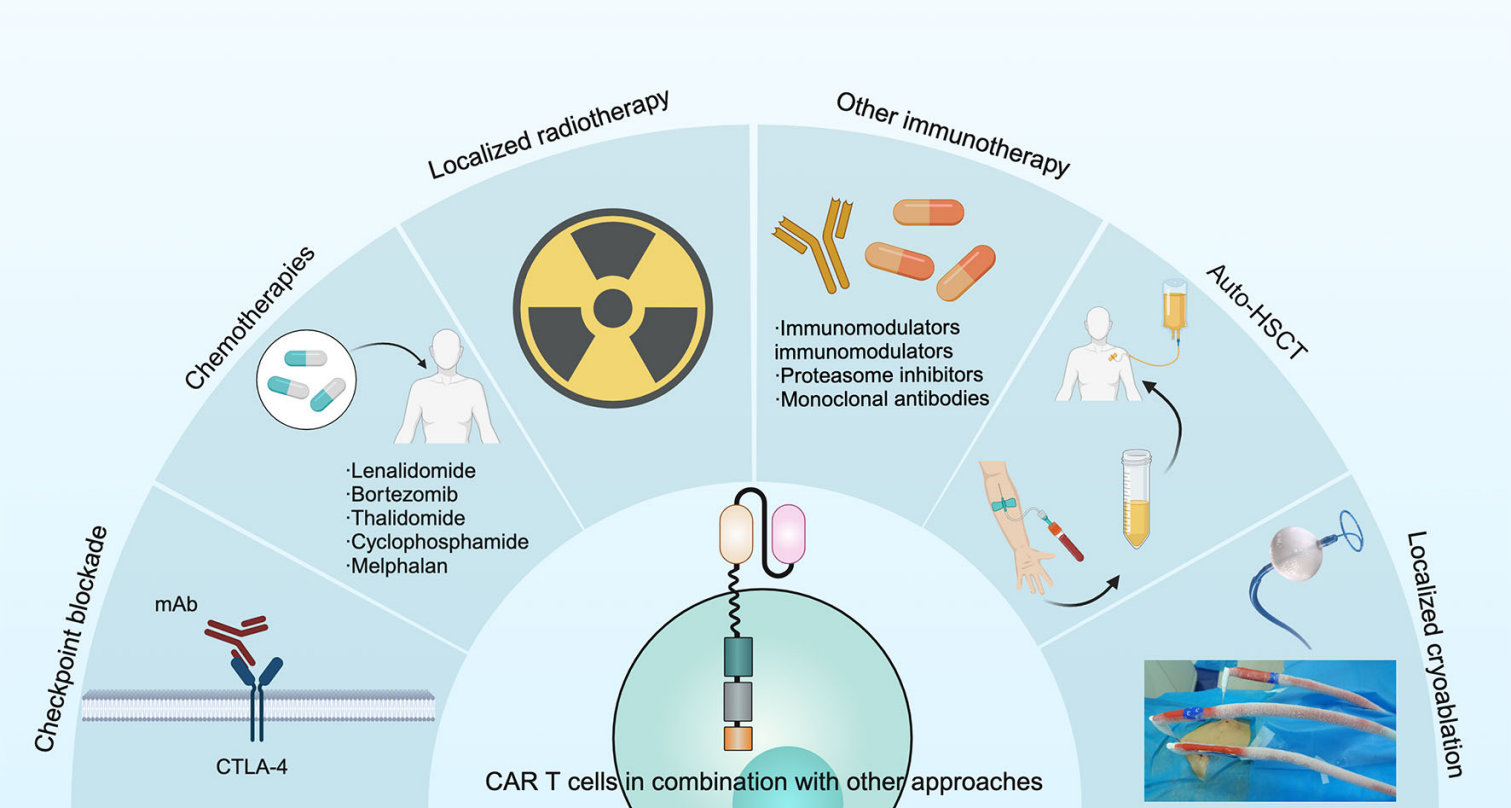
Several approaches have been used in combination with CAR T cells to improve treatment outcomes in MM, including checkpoint blockade, chemotherapies, localized radiotherapy, other immunotherapies, auto-HSCT and localized cryoablation.

Another approach to overcome antigen loss following CAR T cell therapy is co-administration of chemotherapeutic agents (97), checkpoint inhibitors (98), localized radiotherapy, vaccines (99), other immunotherapies (100), autologous hematopoietic stem cell transplantation or localized cryoablation (2). This approach could lead to epitope spreading, which may counteract immune escape (**Figure 6**). Therefore, optimizing the CAR structure and integrating it with other therapies could prove to be a proficient strategy to combat antigen evasion and enhance the efficacy of tumor suppression.

## 6 Future of CAR T cell therapy

The future of CAR T-cell therapy is promising, with ongoing advancements focused on enhancing efficacy by targeting a broader range of cancers, reducing toxicity, developing off-the-shelf solutions, combining with other therapies, expanding applications to various conditions, and improving manufacturing scalability, all of which aim to make it more accessible and effective in personalized treatment.

## 7 Conclusions

CAR T cell therapy represents a new approach to the treatment of MM. Various target antigens, including BCMA, CD19, CD38, CD44v6, CD138, GPRC5D, kappa light chain, FcRH5, CS1, integrin β7, and NKG2D, have been used to engineer CAR T cells to kill myeloma cells, with encouraging results. However, CAR T cell therapy for MM still faces many challenges, such as tumor antigen escape, CRS, ICANS, an inhibitory tumor microenvironment and poor persistence of T cells *in vivo*. Accordingly, researchers have proposed many strategies to optimize CAR T cells, including updating the structure of CARs, improving the tumor microenvironment, promoting the durability of CAR T cells and increasing the safety of CAR T cells. In short, CAR T cell therapy is a novel, alternative and promising treatment for MM.
